# Complexity and specificity of experimentally induced expectations in motion perception

**DOI:** 10.1186/1471-2202-14-S1-P355

**Published:** 2013-07-08

**Authors:** Nikos Gekas, Matthew Chalk, Aaron R Seitz, Peggy Seriès

**Affiliations:** 1 IANC School of Informatics, University of Edinburgh, Edinburgh, EH8 9AB, UK; 2 Department of Psychology, UC Riverside, Riverside, CA, USA

## 

A growing body of work suggests that perception is akin to Bayesian Inference [1]. In this context, it is critical to understand how priors are formed, what complexity of the prior distributions can be learned, and whether learned priors transfer to similar stimuli. Only a handful of studies have started to explore these issues (e.g. [2,3]).

In [4], we had shown that the statistics of past visual motion stimuli can powerfully modulate the perception of new motion directions. Human observers were presented with low contrast white random dots, and were asked to perform a dual task of direction estimation and motion detection. We manipulated subjects' expectations by using a bimodal distribution of motion directions such that two directions were more frequently presented than the others. Subjects were found to quickly and implicitly learn about the stimulus distribution, and their perception was altered in two ways: 1) they perceived motion directions as being more similar to the most frequent directions than they really were, and 2) in the absence of stimuli, the most frequent stimuli were frequently perceived.

We here modify this paradigm to explore whether subjects can learn multiple stimulus distributions simultaneously. We interleaved moving dot displays of two different colors, either red or green, with different motion direction distributions. When one distribution was uniform and the other bimodal, we found that participants learned the statistics of the combined (bimodal) distribution and used that knowledge in both color conditions. When one distribution was bimodal and the other was the vertically flipped image of the first one so that the combined distribution was uniform, we found that participants tried to learn the statistics of each distribution but did not clearly apply that knowledge only to the appropriate condition.

Our findings suggest that it is possible to learn the joint statistics of the stimuli by using a feature like color as a distinguishing factor but only under specific conditions. Even so, there is a tendency for subjects to learn a complex combination of the two distributions and use it non-specifically for the different conditions. Our results can be modeled using a Bayesian framework and discussed in terms of a sub-optimality of the statistical learning process under some conditions. Understanding the limitations of statistical learning for complex stimuli may help understanding how expectations are learned at the neural level.
